# A postnatal peak in microglial development in the mouse hippocampus is correlated with heightened sensitivity to seizure triggers

**DOI:** 10.1002/brb3.403

**Published:** 2015-11-11

**Authors:** Iris Kim, Lauren M. Mlsna, Stella Yoon, Brandy Le, Songtao Yu, Dan Xu, Sookyong Koh

**Affiliations:** ^1^ Ann and Robert H. Lurie Children's Hospital of Chicago Stanley Manne Children's Research InstituteDepartment of PediatricsFeinberg School of MedicineNorthwestern UniversityChicagoIllinois

**Keywords:** Early life, epilepsy, febrile seizures, status epilepticus

## Abstract

**Background:**

Explosive synaptogenesis and synaptic pruning occur in the hippocampus during the first two weeks of postnatal life, coincident with a heightened susceptibility to seizures in rodents. To determine the temporal correlation between microglial development and age‐dependent susceptibility and response to seizures, we quantified developmental changes in basal microglia levels and seizure‐induced microglial activation in the hippocampus of Cx3Cr1^GFP^ ^/+^ transgenic mice.

**Methods:**

Basal levels of microglia were quantified in the hippocampi of Cx3Cr1^GFP^ ^/+^ mice at P0, P5, P10, P15, P20, P25, P30, P40, and P60. Seizure susceptibility and seizure‐induced microglial activation were assessed in response to febrile seizures (lipopolysaccharide followed by hyperthermia) and kainic acid‐induced status epilepticus.

**Results:**

The density of microglia within the hippocampus increased rapidly after birth, reaching a peak during the second week of life – the age at which the animals became most vulnerable to seizure triggers. In addition, this peak of microglial development and seizure vulnerability during the second postnatal week represented the time of maximal seizure‐induced microglia activation.

**Conclusions:**

Overreactive innate immunity mediated by activated microglia may exacerbate acute injury to neuronal synapses and contribute to the long‐term epileptogenic effects of early‐life seizures. Anti‐inflammatory therapy targeting excessive production of inflammatory mediators by activated microglia, therefore, may be an effective age‐specific therapeutic strategy to minimize neuronal dysfunction and prevent increases in susceptibility to subsequent seizures in developing animals.

## Introduction

The rapidly developing brains of infants and children are especially susceptible to seizures. Status epilepticus (SE) – prolonged or repeated seizures lasting longer than 30 min – has the highest incidence in infants less than one year (Hesdorffer et al. 1998). In addition, febrile seizures (FS) occur predominantly between three months and six years of age (Stafstrom 2002), and several epilepsy syndromes resolve after childhood (Brorson and Wranne 1987). It has long been suspected that inflammation and the immune system play a fundamental role in epilepsy. Infectious and autoimmune diseases are often accompanied by recurrent seizures (Dalmau et al. 2008; Kim et al. 2009). Pro and anti‐inflammatory molecules are upregulated in the brain at the sites of seizure initiation and propagation and may influence seizure susceptibility (Murashima et al. 2008; Fukuda et al. 2009; Galic et al. 2012; Choy et al. 2014; Vezzani 2014). Furthermore, microglia – the resident innate immune cells of the central nervous system – are markedly activated in the brains of individuals with chronic intractable epilepsy and in animal models of seizures (Beach et al. 1995; Yang et al. 2000; Drage et al. 2002; Avignone et al. 2008; Choi et al. 2009; Somera‐Molina et al. 2009; Fabene et al. 2010). However, the contribution of the innate immune system to the specific vulnerability of the young brain to seizures has not been well‐defined.

Given their role as the brain's resident immune effectors, microglia are natural targets of investigation for questions about the relationship between immune activity and early‐life seizure susceptibility. In rats, microglia are present throughout the hippocampus by early in the second postnatal week (Dalmau et al. 1998), and differentiation of microglia into mature forms appears to be fully complete by the third week of life (Wu et al. 1992; Streit and Xue 2009). Additionally, emerging evidence for the integral role played by microglia in developmental synapse maturation and elimination suggests that they not only are present, but are functionally active by this time (Stevens et al. 2007; Tremblay et al. 2010; Paolicelli et al. 2011; Schafer et al. 2012). The relationship between early postnatal microglia development and vulnerability to seizure triggers, as well as seizure‐induced microglia activation, remain to be fully characterized. The aim of the present study was to determine the specific age during postnatal development at which microglia reach maximal levels within the hippocampus and to determine whether a relationship exists between the peak of microglia proliferation and heightened vulnerability and responsiveness to seizures.

## Materials and Methods

### Animals

Cx3Cr1^GFP/+^ transgenic mice were obtained as a gift from Dr. Jaime Grutzendler (New Haven, CT). The fractalkine receptor in these mice has been replaced by a green fluorescent protein (GFP) reporter gene by targeted deletion via homologous recombination in embryonic stem cells (Jung et al. 2000; Davalos et al. 2005). Jung et al. (2000) have previously shown that insertion of GFP into the Cx3Cr1 locus does not alter the size of various GFP‐labeled cell populations, including microglia, dendritic cells, and monocytes. Furthermore, an immune challenge to Cx3Cr1^GFP/+^ mice elicits a microglial response indistinguishable from that observed in Cx3Cr1^GFP/GFP^ or WT mice in terms of kinetics, proliferation, differentiation, and number of recruited cells. Mice were group housed in polypropylene cages and maintained at 21°C with ad libitum access to water and rodent chow. All procedures were conducted in accordance with the National Institutes of Health Guidelines for the Care and Use of Laboratory Animals and were approved by the Stanley Manne Children's Research Institute Institutional Animal Care and Use Committee.

### Developmental study

In order to characterize developmental changes in basal microglia levels Cx3Cr1^GFP/+^ mice were sacrificed at the following ages: postnatal day (P) 0, P5, P10, P15, P20, P25, P30, P40, and P60 (*n *=* *3–4/group). Mice were deeply anesthetized via CO_2_ inhalation and perfused transcardially with cold phosphate‐buffered saline (PBS) followed by 4% paraformaldehyde (PFA)/0.1 M sodium phosphate buffer. Harvested brains were further fixed with 4% PFA/4% sucrose solution overnight and mounted on a freezing microtome to obtain 40 *μ*m sections in the coronal orientation. Every sixth section was collected and processed.

### Febrile seizure susceptibility

Our laboratory has developed a new experimental model of FS by combining lipopolysaccharide (LPS), a Toll‐like Receptor 4 (TLR4) ligand, with hyperthermia‐induced seizures in immature mice (Eun et al. 2015; Radzicki et al. 2013). We used this murine model of FS to investigate the age‐dependent behavioral and microglial response to febrile seizures. P5, P10, P15, and P20 Cx3Cr1^GFP/+^ mice (*n *=* *4–11/group were injected with LPS (100 *μ*g/kg; *Escherichia coli*, serotype 0127:B8; Sigma Chemicals Co., St. Louis, MO) intraperitoneally (*i.p*.) 2 h prior to seizure induction and placed in an incubator maintained at an ambient temperature of 30°C. Littermate controls (no seizure, normothermic) were injected with PBS and kept in the same incubator for the duration of the experiment. To induce hyperthermic seizures, mice were placed inside a Plexiglas chamber heated by a heat lamp positioned 10 cm above the chamber. Core body temperature (*T*_b_) was measured using a rectal probe connected to a rodent temperature controller (BAT‐7001H, Physitemp). *T*_b_ was recorded at the beginning of the experiment and every 2 min thereafter for a total of 30 min. Behavioral changes and latency to the first sign of seizure were also monitored. If an animal's temperature reached >41.5°C, it was removed from the Plexiglas chamber and placed on a cool metal surface for 2 min and until T_b_ returned below 41.0°C. Following 30 min of hyperthermia, animals were cooled to normal T_b_ and rehydrated with saline injection. Animals were permitted to recover with their respective dams overnight. 24 h after seizure induction, animals were sacrificed and their brains harvested as in the developmental study for microglial quantification or immunohistochemistry. A subset of animals from the P15 FS and control groups (*n *=* *3/group) was deeply anesthetized via CO_2_ inhalation and sacrificed by decapitation. Left and right hippocampi were dissected, flash frozen on dry ice, and stored at −80°C for real‐time quantitative PCR (RT‐qPCR).

### Kainic acid‐induced status epilepticus susceptibility

Systemic injection of KA in rodents induces limbic seizures which originate in the CA3 region of the hippocampus (Nadler 1981; Ben‐Ari 1985). These seizures spread from the hippocampus to other limbic structures and, in adult rats, are followed by neuronal loss in selected brain regions, reminiscent of brain damage seen in patients with temporal lobe epilepsy. In order to investigate the age‐dependent behavioral and microglial response to Kainic acid‐induced status epilepticus (KA‐SE), seizures were induced via *i.p*. injection of KA (#K‐1013, A.G. Scientific, Inc., San Diego, CA) as previously described (Hu et al. 1998). P5, P10, P15, P25 and P40 Cx3Cr1^GFP/+^ transgenic mice (*n *=* *3–4/group) received injections of either KA or sterile PBS and were observed for 1–3 h. Latency to the first sign of seizure and seizure severity were recorded. Seizure severity was based on the maximal response achieved on a graded scale from 0 to V as follows: 0 – no response; I – behavioral arrest; II – staring, pawing, and head bobbing; III – clonic jerks, rearing and falling; IV – continuous grade III seizures for longer than 30 min (status epilepticus [SE]); V – death.

Age‐appropriate doses of KA which would cause SE were empirically determined. Comparable to what has been observed in earlier studies with rats (Albala et al. 1984), the dose of KA sufficient to produce SE increased with age. In P5 and P10 mice, 2 mg/kg KA doses consistently induced SE ‐ prolonged seizures longer than 1 h. In P15 mice, 5 mg/kg of KA caused 100% mortality within 20 min of injection and 2 mg/kg produced grade II seizures, while an intermediate dose of 2.5 mg/kg consistently produced both intermittent and continuous grade III seizures (SE). In P25 mice KA injections (25 mg/kg) induced SE in 75% (3/4) of animals. Likewise, in P40 mice, 25 mg/kg of KA was required to trigger SE in 80% (4/5) of animals. One day after seizure induction, all animals were sacrificed and their brains harvested for quantification of microglia as in the developmental study. A subset of animals from the P15 KA and control groups (*n *=* *6/group) was deeply anesthetized via CO_2_ inhalation and perfused transcardially with cold PBS. Whole brains were harvested for flow cytometric analysis. For RT‐qPCR, an additional subset of animals from the P15 KA and control groups (*n *=* *3/group) was deeply anesthetized via CO_2_ inhalation and sacrificed by decapitation. Left and right hippocampi were dissected, flash frozen on dry ice, and stored at −80°C.

### Quantification of microglia

To examine changes in the number and size of microglia during development and after FS or KA‐SE, six sections spanning the extent of the dorsal hippocampus were selected from each animal (*n *=* *3–4 animals/group). Left and right hippocampi in each section were examined under fluorescence and values from all hippocampal sections averaged for each animal (*n *=* *12 hippocampal sections/animal). Digital microscopy images were observed and captured at 4 ×  and 20 ×  magnification. For quantification analysis, all GFP‐positive labeled microglia cells in the CA3 subregion of the hippocampus below the pyramidal cell layer were selected at a constant threshold value for all specimens within comparison groups. The percent area of fluorescence was measured using 20× magnification (0.27 mm^2^ area) with Image J64 (1.43u, Public Domain, NIH, RRID:nif‐0000‐30467). For cell counts, the total number of microglia contained in a 0.27 mm^2^ area below the pyramidal cell layer of the CA3 subregion of the dorsal hippocampus was counted manually, excluding cells outside of the CA3 region of the hippocampus for P0 and P5. Similarly, for quantification of soma size, each cell within the 0.27 mm^2^ area captured at 20× magnification was selected manually and the area of fluorescence measured using Image J64. To examine changes in cell morphology during development and after seizures, the CA3 area of hippocampi was observed using confocal fluorescence microscopy at 40× magnification.

### Detection of mRNA levels of *Tnf‐α, Il‐1β*, and inducible nitric oxide synthase using real‐time quantitative PCR

To assess seizure‐induced changes in inflammatory genes following FS and KA seizures, mRNA levels of three inflammation‐related genes were quantified in hippocampi harvested 0 h and 4 h after the onset of FS and KA at P15 and in their control littermates. These time points were chosen because previous work showed cytokine gene induction after FS to be immediate and short‐lived, with blood levels of IL‐1*β*, IL‐6, and TNF‐*α* peaking within 2 h and declining to baseline levels as early as 4 h after FS (Eun et al. 2015). An initial screen of 7 cytokines (IL‐1*β*, IL‐12p70, IFN‐*γ*, IL‐6, KC/GRO, IL‐10, and TNF‐*α*) using an ELISA‐based commercially available kit (Meso‐Scale Discovery, Gaithersburg, MD) revealed significant increases in IL‐1*β* and TNF‐*α* blood cytokine levels after FS (Eun et al. 2015). We therefore chose in our qPCR analysis two proinflammatory cytokines, IL‐1*β* and TNF‐*α*, and added nitric oxide synthase (NOS‐2), an inducible gene involved in the inflammatory cascade, in order to show a functional role of activated microglia prior to morphological changes at 24 h. Total RNAs were isolated from frozen hippocampal samples using TRIzol Reagent (Invitrogen, Carlsbad, CA) following the manufacturer's manual. The total RNAs in each group were pooled and a total of 1 *μ*g of pooled total RNAs were then reverse‐transcribed into cDNA using iScript^™^ cDNA Synthesis Kit (Bio‐Rad, Hercules, CA) according to the manufacturer's protocol. The qPCR primers were as follows: *Tnf‐α*, forward: 5′‐CTCCAGGCGGTGCC TATG‐3′, reverse: GGGCCATAGAACTGATGAGAGG‐3′; *Il‐1β*, forward: 5′‐GCACACCC ACCCTGCA‐3′, reverse: 5′‐ACCGCT TTTCCATCTTCTTCTT‐3′; and *Nos2*, forward: 5′‐TTCCAGAATCCCTGGACAAG‐3′, reverse: 5′‐GGTCAAACTCTTG GGGTTCA‐3′(Ramaglia et al. 2012). 18S rRNA was also amplified to serve as an internal control using the primers as follows: 18S rRNA, forward: 5′‐CGGCTACCACATCCAAGGAA‐3′, reverse: 5′‐AGCCGCGGTAATT CCAGC‐3′(Hazarika et al. 2007). The qPCR reaction was set up using SsoAdvanced^™^ SYBR^®^ Green Supermix and ROX passive dye (Bio‐Rad); each qPCR reaction contained 10 *μ*L of 2X Supermix, 1 *μ*L of each forward and reverse primer (10 *μ*mol/L), 0.4 *μ*L of ROX dye (50×), and approximately 25 ng of cDNA template (2.6 *μ*L). The total volume of reaction was adjusted to 20 *μ*L with molecular grade H_2_O. qPCR was performed on Applied Biosystems 7500 Fast Real‐Time PCR System (Life Technologies, Foster City, CA) under the following conditions: 95°C for 5 min, 40 cycles at 95°C for 10 sec, and 60°C for 45 sec, followed by a dissociation stage. The relative gene expression was finally determined using the ∆∆*C*_t_ method.

### Microglia double‐labeling and colocalization analysis

In order to verify colocalization of the Cx3Cr1^GFP/+^ signal with the microglial marker, IBA1, hippocampal sections from brains harvested 24 h after FS at P15 and their control littermates were immunostained for IBA1. IBA1, also known as Aif1, has been shown to preferentially label activated over resting microglia (Postler et al. 2000; Ito et al. 2001; Schwab et al. 2001).

#### Immunohistochemistry

Forty micrometer sections were rinsed with PBS and nonspecific binding sites were blocked in PBS containing Triton‐X (0.1%), normal rabbit serum (5%), and bovine serum albumin (2%) for 1 h at 23**°**C. Slices were incubated overnight at 4**°**C with primary anti‐IBA1 (1:200, Abcam, Cat# ab5076, RRID:AB_2224402) in PBS containing bovine serum albumin (0.2%). Primary antibody binding was amplified and visualized with Alexa 594‐conjugated rabbit anti‐goat antibody (1:500, Invitrogen, Cat# A21223, RRID:AB_1500720). Coverslips were mounted using Prolong Gold Antifade Reagent (Invitrogen) and observed at 10×, 20×, and 63× magnification.

#### Colocalization analysis

Qualitative analysis of colocalization of IBA1 and Cx3Cr1 was conducted using the ImageJ plug‐in JACoP (Just Another Colocalization Plugin) (http://rsb.info.nih.gov/ij/plugins/track/jacop.html; (Bolte and Cordelieres 2006). Background subtraction was performed to eliminate noise. A correlation of signal intensity was calculated as a Pearson's coefficient. A cytofluorogram was plotted as the normalized intensity of Cx3Cr1 (green) as a function of IBA1 (red).

### Flow cytometric analysis of antigen presenting cells

In order to confirm that a vast majority of Cx3Cr1^GFP/+^ cells within the brain are microglia at 24 h post‐KA‐SE, we performed flow cytometry to characterize different subsets of antigen presenting cells (APCs) in the brain. Whole brains harvested from Cx3Cr1 mice 24 h after KA‐SE at P15 and their PBS control littermates were pooled (*n *=* *6/group) and processed into single‐ cell suspensions. Cells were isolated and stained as previously described (Bailey et al. 2007). Briefly, Fc receptors (FcR) were blocked with *α*CD16/CD32 (BD Biosciences, Cat# 553142, RRID:AB_394657) and cells were stained with various combinations of the following antibodies: *α*Ly6C‐APC (Biolegend, Cat# 128016, RRID:AB_1732076), *α*CD11c‐APC/Cy7 (Biolegend, Cat# 117324, RRID:AB_830649), *α*CD11b‐BV421(Biolegend, Cat# 101236, RRID:AB_11203704), *α*CD45‐V500 (BD, Cat# 561487, RRID:AB_10697046), *α*CD3‐PE (Biolegend, Cat# 100308, RRID:AB_312673), *α*CD39‐PE/Cy7 (Biolegend, Cat# 143806), *α*Ly6G‐PerCP/Cy5.5(Biolegend, Cat# 127616, RRID:AB_1877271), *α*B220‐Alexa700 (BD, Cat# 557957, RRID:AB_396957) and Live‐dead blue (Invitrogen, Cat# L‐23105). Flow cytometric data were collected on a FACS Fortessa flow cytometer (BD Biosciences, San Jose, CA) using BD FACSDiva (BD Biosciences, RRID:SciRes_000115) and analyzed with FlowJo software (Tree Star, Ashland, OR, RRID:nif‐0000‐30575).

### Statistical analysis

Student's (unpaired) *t*‐tests (GraphPad Prism v. 5.0, GraphPad Software Inc., San Diego, CA, RRID:rid_000081) were used to compare the latency to seizure onset, expression levels of inflammation‐related genes, and quantification of microglial activation. One‐way analysis of variance (ANOVA) with a post hoc *t*‐test and Tukey corrections were used to compare differences among different experimental groups. Fisher's exact test was used to compare the proportion of animals experiencing FS between different age groups. Values are expressed as mean ± standard error of the mean (SEM). Significance was defined as *P *<* *0.05 for all tests.

## Results

### Hippocampal microglia development peaks at P10–P15

**Figure 1:**
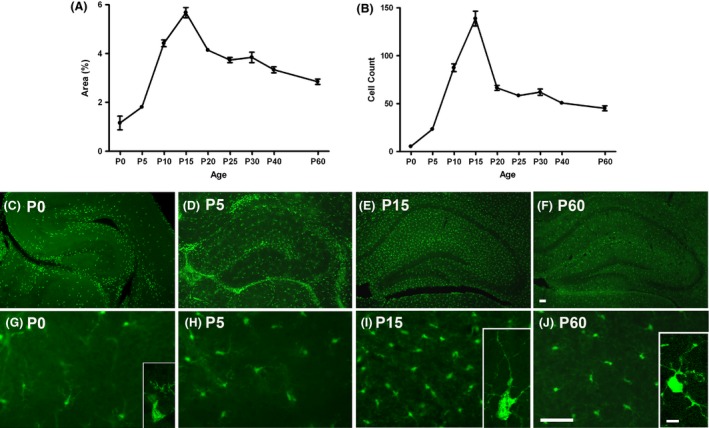
Developmental profile of microglia in mouse hippocampus. A steep rise in microglia occurs in the hippocampus from P0 to P15. The sharpest increase is observed from P10 to P15. There is an abrupt decrease in percent area of fluorescence and in the number of microglia from P15 and P20. (A) Quantification of the mean percent area of Cx3Cr1‐GFP fluorescence above threshold within the CA3 subregion of the hippocampus (*n *=* *3–4/age). (B) Cell counts of microglia within CA3 subregion. (C–J) Representative images of GFP‐labeled microglia in coronal sections of the right hippocampus of P0, P5, P15 and P60 mice. (C–F) Upper panel: 4 ×  (hippocampus); (G–J) Lower panel: 20× (CA3). Scale bars = 50 *μ*m. (Inset, G I &J) Representative confocal images of individual microglia within the hippocampus. Small cell bodies and poor ramification are noted at P0. At P15, microglia have larger bodies with elaborate processes extending into the hippocampal parenchyma. Microglia at P60 demonstrated smaller somas and fine, ramified branches typical of mature, resting microglia. Scale bar = 25 *μ*m.

At P0, the hippocampus was nearly devoid of microglia (Fig.**** 1). While a slightly increased number of cells was found within the hippocampus at P5, a dramatic upsurge of microglia occurred by P10 and P15. There was a sixfold increase in the number of fluorescent cells in the hippocampus between P5 and P15 (Fig. 1B). Microglia exhibited the highest density and were evenly distributed throughout the hippocampus at P15. The percent area of fluorescence and cell count declined substantially from P15 to P20, and decreased steadily thereafter from P25 to P60 (Fig. 1A,B). A higher magnification view of hippocampal microglia (Fig. 1, Insets) shows that P15 microglia bear well‐defined, elongated processes that are absent shortly after birth (P0) but comparable to those of fully developed microglia at adulthood (P60).

### Seizure susceptibility is correlated with the peak of microglia development in the hippocampus

#### Febrile seizures

**Figure 2:**
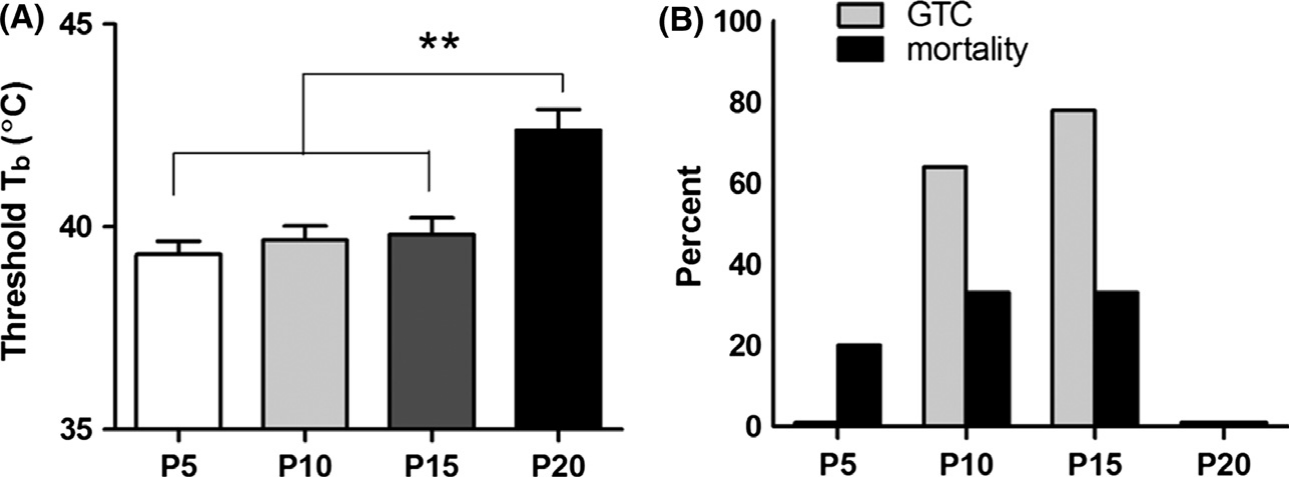
Age‐dependent response of postnatal mice to FS. (A) Mean threshold core body temperature (*T*_b_) at first sign of seizure (rapid tail shaking). Threshold temperatures at seizure onset were significantly lower in P5, P10, and P15 mice (39.3 ± 0.7°C, 39.7 ± 1.2°C, and 39.9 ± 1.2°C, respectively) compared to P20 mice (42.4 ± 0.3°C) (*n *=* *4–11/group; one‐way ANOVA:* P *<* *0.01; Tukey post hoc tests: **P <* *0.01). Each bar represents the mean ± SEM. (B) Percentage of animals experiencing generalized tonic‐clonic (GTC) seizures and percent mortality. Mice at the second week of life had the highest frequency of GTC seizures: 64% of P10 animals (*n *=* *11) and 78% of P15 animals (*n *=* *9). No single animal at P5 (*n *=* *5) or P20 (*n *=* *4) had GTC seizures. FS‐induced mortality was greatest among P10 and P15 mice (both at 33%).

There was an age‐dependent behavioral response to FS (Fig.**** 2). P5 animals displayed rapid tail shaking as the initial sign of seizure at an average threshold temperature of 39.3°C, followed by head bobbing and loss of postural control (Fig. 2A). The pups then exhibited clonic movements of hind limbs, which transitioned into flailing behavior involving clonic movements of all extremities. In P10 and P15 animals, seizures began with tail shaking (similar to P5 animals) at 39.7°C and 39.9°C, respectively. Unlike P5 animals, however, the seizures then evolved to generalized tonic‐clonic (GTC) convulsions characterized by rigid flexion of the trunk and continuous, rapid, and synchronous movements of the face and limbs. The average threshold temperature for GTC seizures was 43.0 ± 0.1°C. Threshold temperatures for initial sign of seizures or for GTC seizures were similar between P10 and P15, and a comparable proportion of P10 and P15 animals experienced GTC seizures (64% vs. 78%, *P *=* *0.64, Fisher's exact test). P20 animals showed seizure behavior at 42.4°C but did not proceed beyond tail shaking and clonic movements of individual limbs, even at core temperatures as high as 43.4°C. All animals in this age group survived (Fig. 2B).

**Figure 3:**
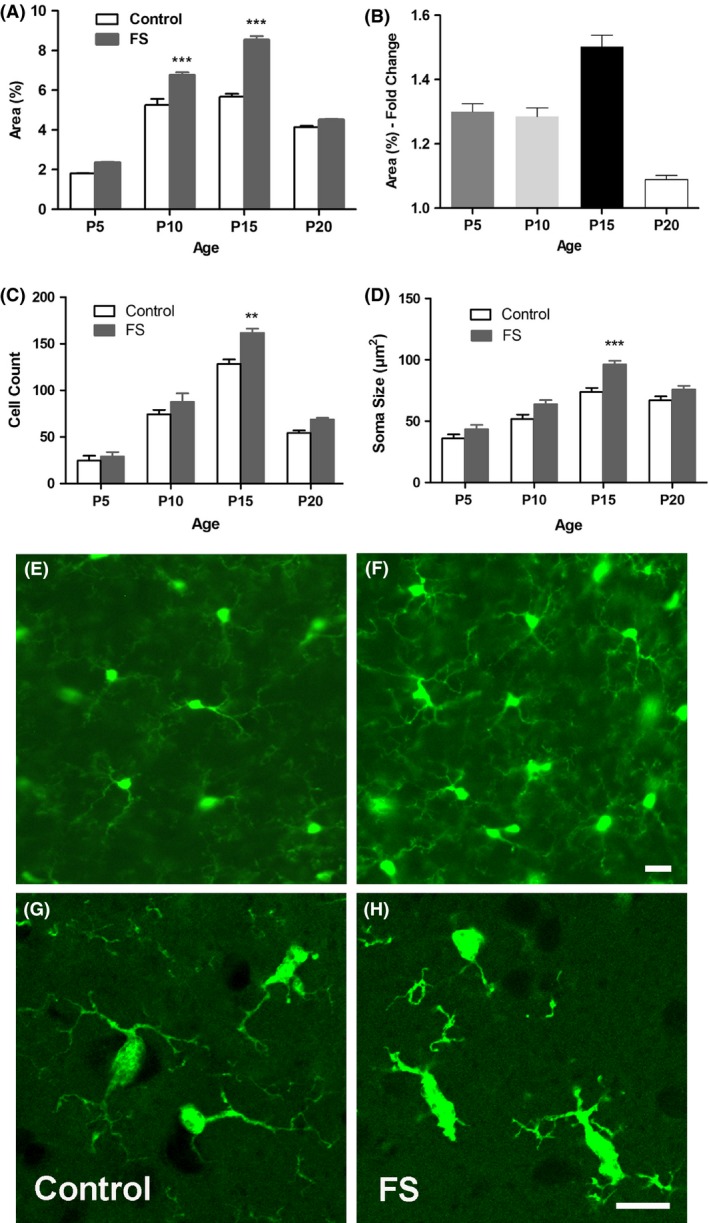
Microglial activation in developing hippocampus 24 h after FS. (A) Quantification of FS‐induced increase in percent area fluorescence in P5, P10, P15 and P20 mice. FS led to significant microglia activation at P10 and P15. (B) The greatest fold change in percent area of microglial fluorescence after FS occurs at P15. Each bar represents the mean ± SEM (*n *=* *3–4/group; one‐way ANOVA:* P *<* *0.0001; Tukey post hoc comparisons: ***P <* *0.001). Increase in (C) number and (D) soma size of Cx3Cr1‐GFP+ microglia after FS (*n *=* *3/group; one‐way ANOVAs: *P *<* *0.0001; Tukey post hoc comparisons: **P <* *0.01, ***P <* *0.001). (E‐H) Representative images of microglia in the CA3 subregion at P15 showing robust activation of microglia. (E,G) Control; (F, H) FS. Confocal microscopy shows activated microglia in (H) with brighter and larger cell bodies and thicker and shortened processes after FS. Scale bars = 25 *μ*m.

Similar to the variations in seizure behavior, the degree of microglia activation after FS differed by age (Fig.**** 3A and B). P5 and P10 mice showed only a modest level of microglia activation after severe seizures. While P10 and P15 mice had similar basal quantities of hippocampal microglia, P15 mice showed a greater increase in the % area of fluorescence after seizures. FS produced only a slight level of activation at P20. At P15, but not at P5, P10, or P20, the increase in percent area fluorescence was accompanied by an increase in cell numbers and soma size after FS (Fig.**** 3C and D). Therefore, microglia activation 24 h after FS was most intense at P15, with a greater than 1.5‐fold increase in percent area of fluorescence (Fig.**** 3E–H).

#### Kainate‐induced status epilepticus

**Figure 4:**
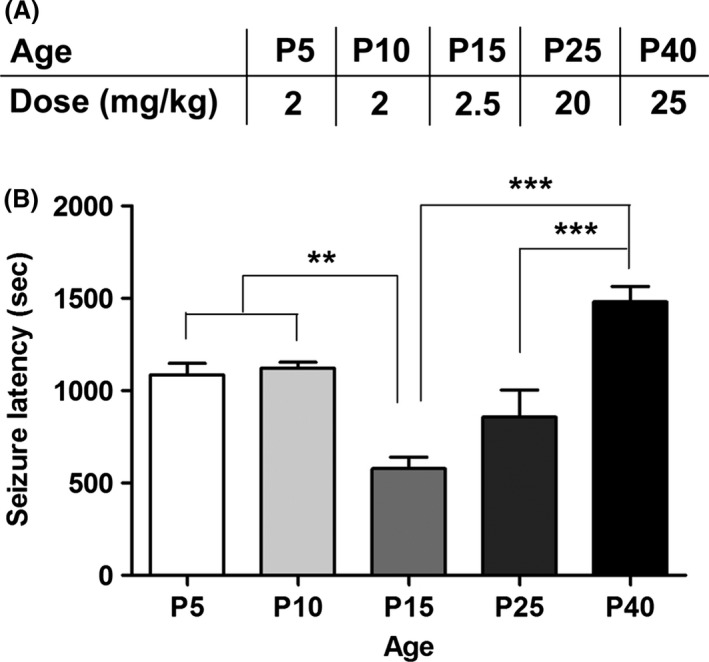
Maturation‐dependent sensitivity to KA. (A) Dose of KA used to elicit status epilepticus. (B) Latency to seizure following *i.p*. injection of PBS or KA in P5, P10, P15, P25, and P40 mice. Latency to seizure was shortest in the P15 group, indicating greatest seizure susceptibility. Latency to seizure was longest in the P40 group. (*n *=* *3–4/group; one‐way ANOVA:* P *<* *0.0001; Tukey post hoc comparisons: **P <* *0.01; ***P <* *0.001; P5/P10 compared to P40 *P *<* *0.05).

P15 mice showed exquisite sensitivity to KA, requiring only 1/8th of the dose necessary for mice at P25 to achieve the same severity of seizures (Fig. 4A). Mice at P15 had the shortest latency to seizure, which manifested as head jerks, forelimb or hindlimb clonus, or rapid tail tremors (Fig. 4B). In P40 mice, a significant delay in seizure onset was noted compared to younger mice, despite requiring the highest dose of KA to induce seizures.

**Figure 5:**
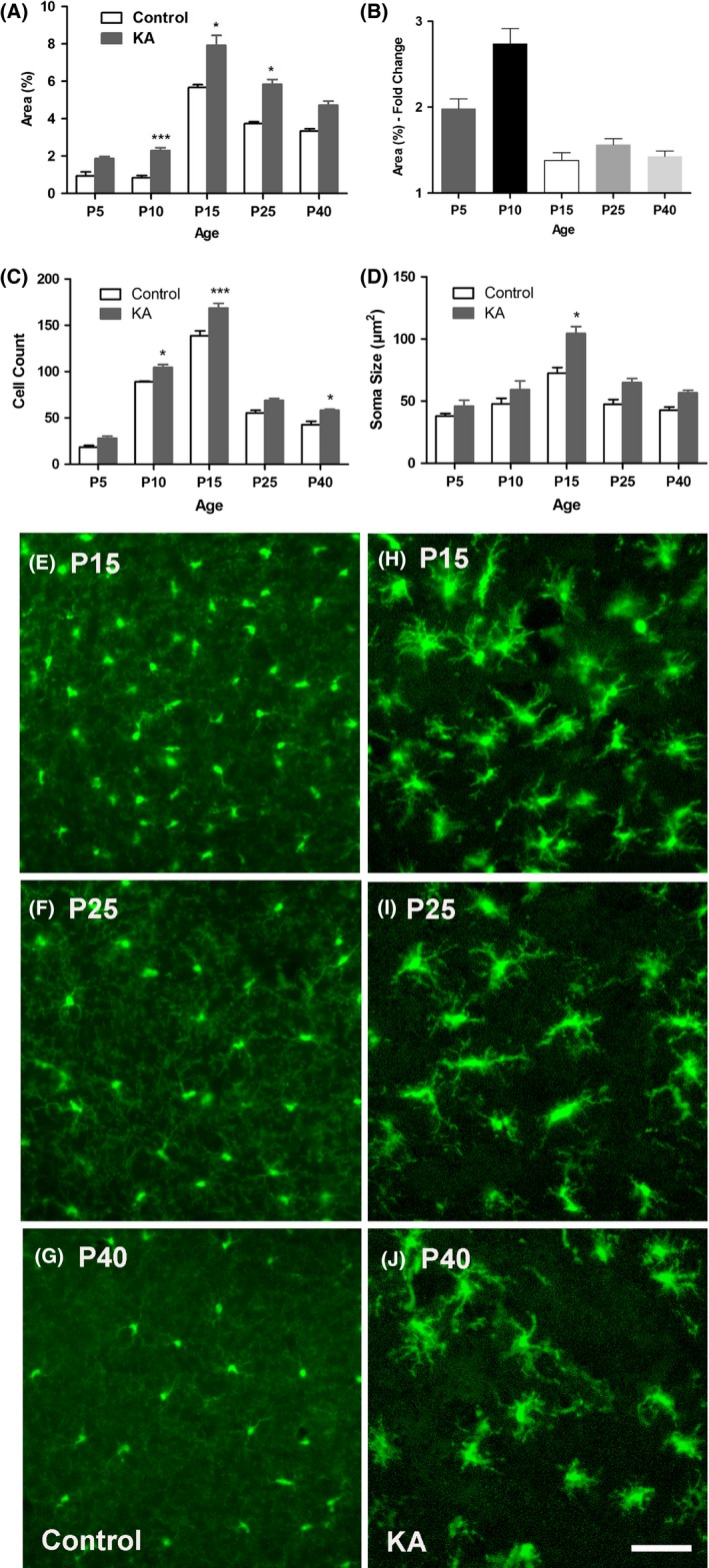
KA–SE‐induced microglia activation. (A) Quantification of microglia activation 24 h after KA‐SE shows a significant seizure‐induced increase in percent area fluorescence at P10, P15 and P25. (B) The greatest fold change in percent area of microglial fluorescence after KA‐SE occurs at P10. Each bar represents the mean ± SEM *(n *=* *3/group; one‐way ANOVA:* P *<* *0.0001; Tukey post hoc comparisons: *P <* *0.05, ***P <* *0.001). Increase in (C) number and (D) soma size of Cx3Cr1‐GFP+ microglia after KA (*n *=* *3/group; one‐way ANOVAs: *P *<* *0.0001; Tukey post hoc comparisons: *P <* *0.05, ***P <* *0.001). (E–J) Representative images of microglia in the hippocampal CA3 subregion showing marked microglia activation. (E–G) Left panel: control; (H–J) Right panel: KA. All images were captured under the same magnification (20×). Scale bars = 50 *μ*m. KA‐SE caused engorgement of somas and increased numbers of retracted and thickened processes. Fine, web‐like green background staining of ramified processes in control animals is no longer observed after KA‐SE.

Dramatic microglial activation occurred in the hippocampus of mice following KA‐SE at P5, P10, P15, P25 and P40 (Fig. 5A and B). P15 mice showed the greatest percent area of fluorescence following KA‐SE, partly due to higher baseline levels of microglia at P15 (Fig. 5E and H). Likewise, P15 animals showed the most exaggerated change in microglia cell numbers (Fig. 5C) and soma size (Fig. 5D), and exhibited enlarged cell bodies and numerous thickened processes.

### Inflammation‐related genes are upregulated after seizures

**Figure 6:**
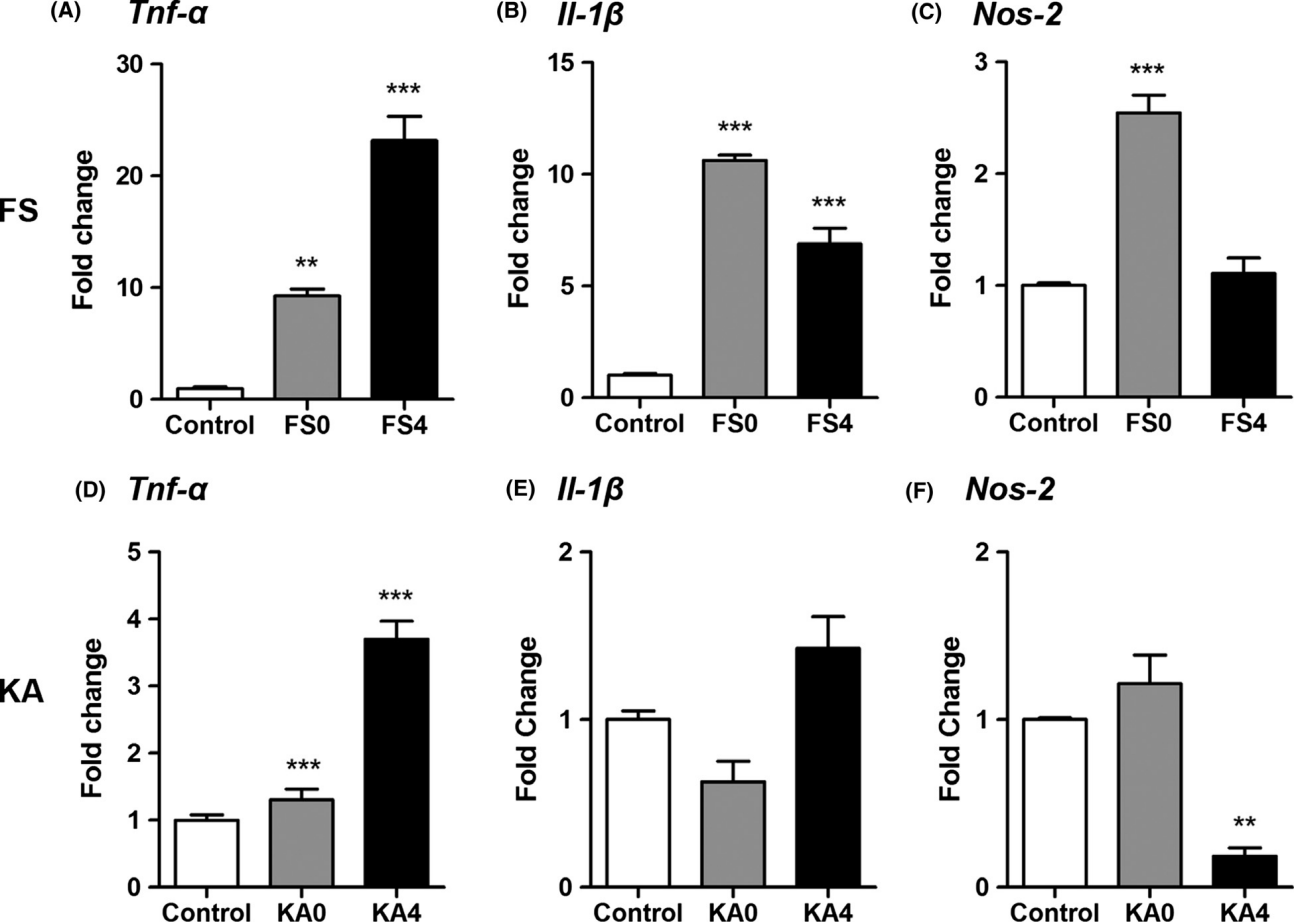
Altered expression of proinflammatory transcripts after FS and KA‐SE. (A, B) Levels of *Tnf‐α* and *Il‐1b* mRNA were significantly higher both immediately after 30 min exposure to hyperthermia (FS0) and 4 h after FS (FS4) than in controls. (C) Expression levels of *Nos2* mRNA were markedly increased at FS0 but returned to baseline levels by FS4. (*n *=* *3/group; one‐way ANOVAs for all genes: *P *<* *0.0001; Tukey post hoc comparisons: **P <* *0.01, ***P <* *0.001). FS0 = immediately following 30 min of hyperthermia and 2.5 h after LPS injection. FS4 = 4 h after hyperthermia and 6.5 h after LPS injection. (D) Levels of *Tnf‐α* mRNA were significantly increased both 30 min (KA0) and 4 h (KA4) after the onset of KA‐induced convulsive seizures compared to controls. In contrast, *Il‐1β* (E) and *Nos2* (F) did not increase significantly after KA seizures (*n *=* *3/group; one‐way ANOVA:* Tnf‐α* and *Nos2*:* P *<* *0.001, *Il‐1β: P *<* *0.05; Tukey post hoc comparisons: **P <* *0.01, ***P <* *0.001). Each bar represents the mean ± SEM.

To further characterize the functional effect of microglia activation after febrile and KA‐SE, RT‐qPCR was used to measure levels of inflammation‐related genes 30 min after the onset of hyperthermia (FS0) or of KA‐induced convulsive seizures (KA0) and 4 h after FS (FS4) or KA‐SE (KA4) in P15 mice (Fig. 6A–F). Expression of *Tnf‐α* and *Il‐1β* and inducible nitric oxide synthase, *Nos2* are known to be upregulated by activated microglia (Hanisch 2002; Smith et al. 2012; Bechade et al. 2014). mRNA levels for each of the genes were significantly increased compared to untreated controls both immediately following and 4 h after FS induction, with the greatest increase in *Tnf‐α* levels (10‐fold). *Tnf‐α* remained elevated (>20‐fold) 4 h after FS and was the only gene that was significantly elevated after KA.

### Cx3Cr1^GFP/+^ are identified as microglia

**Figure 7:**
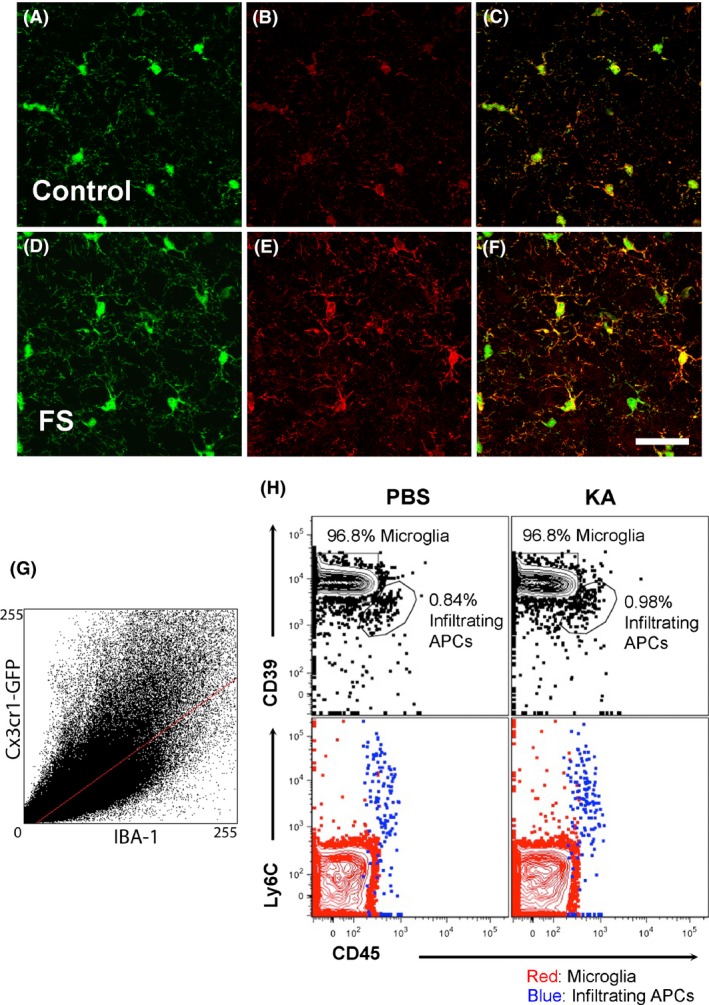
Colocalization of Cx3Cr1‐GFP with IBA1 and flow cytometric analysis of APCs. (A–C) Baseline expression of Cx3Cr1‐GFP (A) and IBA1 (B) by microglia in the CA3 region of the hippocampus 24 h after FS at P15. (D–F) Expression of Cx3Cr1‐GFP (D) or IBA1 (E) by microglia in the CA3 region of the hippocampus after FS at P15. (C, F) Cx3Cr1 and IBA1 are highly colocalized in both conditions. Scale bar = 50 *μ*m. (G) A cytoflurogram of Cx3Cr1‐GFP with RFP‐labeled IBA1 cells 24 h after FS. In P15 control animals, the thresholded Manders’ coefficients for the fraction of Cx3Cr1‐GFP overlapping IBA1 (tM1) and for the fraction of IBA1 overlapping Cx3Cr1‐GFP (tM2) were 0.49 and 0.68, respectively. The Pearson correlation coefficient (Rr) was 0.67. In mice that experienced FS at P15, tM1 = 0.67, tM2 = 0.77, and Rr = 0.78. (H) Flow cytometric analysis of brain‐resident microglia versus brain‐infiltrating macrophage/monocytes 24 h after KA‐SE. The frequencies of microglia (red) and infiltrating macrophage/monocytes (blue) are shown for PBS controls (left column) and KA‐SE (right column). A combination of markers CD39/CD45/Ly6C was used to distinguish these two cell types. Microglia express high levels of CD39, but low levels of Ly6C and CD45 (CD39^hi^ CD45^low^Ly6C^low^), while macrophage/monocytes are CD39^int^ CD45^hi^Ly6C^hi^. Microglia and macrophage/monocytes were gated on forward scatter/side scatter (FSC/SSC) appropriate for antigen presenting cells that were live and CD11b^+^/ CD11c^−^/ CD3^−^/Ly6G^−^/ B220^−^.

We performed a colocalization analysis on hippocampal sections collected from Cx3Cr1^GFP/+^ mice and immunostained for IBA1 in both FS and control groups 24 h after FS at P15 (Fig. 7A–G). There was substantial overlap of GFP‐labeled microglia (green) with IBA1 immunostaining (red) in the CA3 region of the hippocampus both in FS mice (Pearson correlation coefficient [Rr] = 0.78) and in control mice (Rr = 0.67). Because Cx3Cr1 may be expressed not only by microglia but also by other antigen‐presenting cells (APCs: macrophages, monocytes and natural killer cells) flow cytometry was used to determine the frequency of microglia compared to infiltrating APCs within the brain after KA‐SE. 24 h after KA‐SE at P15, at the same time point GFP‐positive cells were quantified (Fig. 7), nearly all of the APCs (excluding dendritic cells) in the brain were microglia (>100:1 microglia: infiltrating macrophage/monocytes) (Fig. 7H). Notably, the ratio was nearly identical in the KA‐SE and PBS control groups.

## Discussion

We found that the number of microglia within the hippocampus progressively increased after birth, reaching a peak during the second week of life. Hippocampal microglia at P10–P15 assumed the highest % area of fluorescence and maximum density, and exhibited the large cell bodies and distinct extension and branching of processes characteristic of fully matured cells. After P15, the density of microglia decreased sharply by P20 and more gradually thereafter as mice approached adulthood. Furthermore, the peak of microglia development we observed at P10–P15 was linked to an exquisite sensitivity to seizure triggers. Animals during the second week of life were not only the most vulnerable to febrile and KA‐induced seizures, but also showed more substantial microglia activation in the hippocampus after seizures than other ages examined.

Previous studies have established that immature rodents are highly susceptible to hyperthermia‐ or hypoxia‐induced seizures during the first two weeks of life (Jensen et al. 1998; Chen et al. 1999; Jensen and Baram 2000; Rakhade and Jensen 2009), and that age‐dependent differences exist in microglial activation following KA‐induced seizures (Rizzi et al. 2003; Ravizza et al. 2005; Jarvela et al. 2011). We demonstrate here that a temporal correlation exists between maximal basal microglia levels in the hippocampus and heightened seizure susceptibility. At P15, FS and KA‐SE‐induced transient upregulation of inflammation‐related genes.

The early postnatal peak in microglia development and responsiveness we observed coincides with a surge of synaptogenesis and synaptic pruning occurring in the hippocampus at this time. Microglia are now known to be key mediators of synapse remodeling and elimination (Wake et al. 2009; Paolicelli et al. 2011; Tremblay et al. 2011). They are also the only cells in the CNS known to express the C3 receptor (Gasque et al. 1998), a complement factor of the classical activation cascade that is implicated, along with C1q, in a mechanism of synapse elimination (Stevens et al. 2007). Since the second postnatal week represents a time of dynamic synaptic pruning by microglia, it makes sense that microglia at P10–P15 are present at high densities in the hippocampus, have achieved a mature phenotype (Perry et al. 1985) and morphology, and would be especially sensitive to activation during this period (Bilbo and Schwarz 2009). It is tempting to speculate that they would be poised to mount an exaggerated inflammatory response against seizures and may lead to excessive synapse elimination of both existing and nascent synapses, which in turn may lead to neuronal dysfunction. Moreover, there is evidence that activated microglia, through the integrin CD11b in cooperation with the immunoreceptor DAP12, trigger developmental apoptosis of hippocampal neurons via the production of superoxide ions (Wakselman et al. 2008). It is therefore possible that seizure‐induced microglia activation could also lead to excessive neuronal death.

The question remains of the mechanism by which the high density of mature microglia present at P10–P15 contribute to a heightened vulnerability to seizure triggers. One possibility is via the actions of proinflammatory cytokines released by activated microglia. Cytokines are not only produced during seizures, but may also contribute to their induction via effects on neuronal excitability. IL‐1*β*, for instance, is rapidly upregulated in microglia after the induction of acute seizures (Vezzani et al. 2010; Jarvela et al. 2011), and receptors for IL‐1*β* are present at high density in the hippocampus, specifically the dentate gyrus (Takao et al. 1990; Ban et al. 1991). Furthermore, IL‐1*β* decreases inhibitory GABA currents and can lead to increases in synaptic excitability (Wang et al. 2000; Rossi et al. 2012). Administration of IL‐1*β* prior to KA increases the time spent in seizures via an NMDA receptor‐dependent mechanism (Vezzani et al. 1999). Reduction of the biologically‐active form of IL‐1*β* by interleukin‐converting enzyme significantly reduces seizure onset and duration (Ravizza et al. 2006), and intracerebral injection of IL‐1 receptor antagonist (ra) produces an anticonvulsant effect (Vezzani et al. 2002). Additionally, mice engineered to overexpress IL‐1ra in astrocytes have decreased seizure susceptibility (Vezzani et al. 2000). Alternatively, microglia may exert effects on neuronal excitability via a cytokine‐independent mechanism. Specifically, microglia express neurotransmitter receptors (Pocock and Kettenmann 2007; Hung et al. 2010) and modulate neuronal activity (Tremblay 2011) indirectly via influences on astrocytes (Pascual et al. 2012). Further studies are needed to elucidate the exact mechanism by which microglia might affect seizure vulnerability.

Finally, an improved understanding of the contribution of microglia development to age‐dependent seizure vulnerability could provide a vital tool in the advancement of age‐specific drug therapies for childhood epilepsy. It is estimated that P0‐5 in rodents corresponds roughly to the third trimester in humans, P7‐10 to the first year of life, and P21 to the transition to the early juvenile period.(Gottlieb A, Keydar Y, Epstein HT. Rodent brain growth stages: an analytical review. Biol Neonate. 1977;32:166–176; Galanopoulou AS & Moshé SL. Pathogenesis and new candidate treatments for infantile spasms and early life epileptic encephalopathies: A view from preclinical studies. *Neurobiol Dis*. 2015; 79:135–149; Akman, O, Moshe SL, Galanopoulou, AS. Sex‐specific consequences of early life seizures. *Neurobio. Dis*. 2014; 72 (Pt B); 153–166 S. Avishai‐Eliner, S, Brunson KL, Sandman, CA, Baram TZ. Stressed‐out, or in (utero)? *Trends Neurosci*., 25; 2002; 518–524.). Our finding showing the high seizure susceptibility between P10 and P25 is consistent with observed heightened vulnerability of infants and young children to seizure triggers. Currently available antiepileptic drugs (AEDs) fail to control seizures in over 30% of pediatric epilepsy patients. Rather than improve behavioral deficits associated with epilepsy or modify disease progression, AEDs themselves can cause cognitive, behavioral, and mood‐altering side effects (Kwan and Brodie 2000; Temkin 2001). Expanding the arsenal to include anti‐inflammatory therapies to target activated microglia would not only represent a novel therapeutic strategy to hasten recovery of neuronal function, but also has the potential to be disease‐modifying and block the epileptogenic effect of prolonged early‐life seizures.

## Conflict of Interest

None declared.
